# Automated Nociceptive Withdrawal Reflex Measurements Reveal Normal Reflex Thresholds and Augmented Pain Ratings in Patients with Fibromyalgia

**DOI:** 10.3390/jcm9061992

**Published:** 2020-06-25

**Authors:** Johannes Ydrefors, Tomas Karlsson, Ulrika Wentzel Olausson, Bijar Ghafouri, Ann-Charlotte Johansson, Håkan Olausson, Björn Gerdle, Saad S. Nagi

**Affiliations:** 1 Department of Clinical Neurophysiology, Linköping University, 58185 Linköping, Sweden; johannes.ydrefors@liu.se (J.Y.); hakan.olausson@liu.se (H.O.); 2 Center for Social and Affective Neuroscience, Linköping University, 58185 Linköping, Sweden; ann-charlotte.johansson@liu.se; 3 Institute of Neuroscience and Physiology, Sahlgrenska Academy, University of Gothenburg, 40530 Gothenburg, Sweden; tomas.karlsson@mednet.gu.se; 4 Pain and Rehabilitation Centre, Department of Health, Medicine and Caring Sciences, Linköping University, 58185 Linköping, Sweden; ulrika.wentzel.olausson@liu.se (U.W.O.); bijar.ghafouri@liu.se (B.G.); bjorn.gerdle@liu.se (B.G.)

**Keywords:** chronic pain, electromyography, fibromyalgia, nociceptive withdrawal reflex, sensitization

## Abstract

The nociceptive withdrawal reflex (NWR) is used to probe spinal cord excitability in chronic pain states. Here, we used an automated and unbiased procedure for determining the NWR threshold and compared the reflex thresholds and corresponding pain ratings in a well-characterized cohort of fibromyalgia (*n* = 29) and matched healthy controls (*n* = 21). Surface electrical stimuli were delivered to the foot in a stepwise incremental and decremental manner. The surface electromyographic activity was recorded from the ipsilateral tibialis anterior muscle. Fibromyalgia patients reported significantly higher scores for psychological distress and pain-related disability and a significantly lower score for perceived state of health compared to the matched controls. The subjective pain ratings were significantly higher in patients. The NWR thresholds were similar to the controls. In the patients, but not in controls, the NWR thresholds and subjective pain ratings were significantly correlated. Our results showed an increased subjective pain sensitivity in fibromyalgia, but we found no evidence for spinal sensitization based on the reflex measures.

## 1. Introduction

The nociceptive withdrawal reflex (NWR) is a polysynaptic spinal reflex evoked by painful cutaneous stimuli. Early studies show that the cutaneous noxious electrical stimulation of the distal lower leg elicits a reflex of muscle contraction and inhibition, resulting in the withdrawal of the limb from the stimulus [1,2,3]. The NWR is thus a protective reflex elicited by potentially harmful stimuli and involves both sensory and motor systems. Further, there is typically an association between the threshold for eliciting the NWR and the subjective pain threshold [4,5]. Based on that, the NWR is considered an objective measure of spinal cord excitability (or sensitization), and an abnormal NWR may reflect changes in the sensory and/or motor systems in different pain disorders [6,7,8,9,10,11].

To elicit the reflex, most studies use the electrical stimulation of the sural nerve distally at the ankle and record the muscle activity of the short head of the ipsilateral biceps femoris muscle [4,7,8,12]. An alternative approach is to stimulate the sole of the foot and record the response of the ipsilateral tibialis anterior muscle [13,14,15,16]. Both methods evoke a withdrawal reaction with the flexion of the knee and ankle joints, but the foot arch-to-tibialis anterior approach is more effective in eliciting the reflex [14].

The reflex afferent is generally presumed to be of the thinly myelinated Aδ type (hence the term RIII reflex, implying the involvement of group III fibers) [17,18]. Several studies have estimated that the reflex latency is approximately 100 ms, which corresponds to the conduction velocity range of Aδ fibers [18,19,20]. A contribution of C fibers to the reflex is also suggested [21,22]. In addition, some studies demonstrate that the NWR might be elicited through the stimulation of thickly myelinated Aβ afferents, at least in certain stimulation paradigms [23]. An early study using the foot sole-to-tibialis anterior approach reported a nociceptive reflex latency of 55 ms, which suggests an afferent signal through fast-conducting Aβ fibers [24]. The involvement of Aβ fibers in nociceptive signal conduction is questioned by others [25]. However, in a recent study, nociceptive signaling by fast-conducting Aβ fibers was demonstrated [26].

Fibromyalgia is characterized by chronic and generalized hyperalgesia according to the American College of Rheumatology (ACR) criteria of 1990 [27], which is often associated with additional symptoms such as sleep disturbance, fatigue, and anxiety [28]. The etiology remains largely unknown, but several hypotheses are proposed, including neuroendocrine and neuroinflammatory processes [29,30]. The pathophysiology is associated with the sensitization of the nociceptive input at both central and peripheral levels [31,32]. In some studies, the NWR threshold is reduced in fibromyalgia, which is consistent with spinal sensitization [7,8,33]. However, other studies show no difference or even a higher threshold for eliciting the NWR in fibromyalgia compared to healthy controls [9,34]. Unlike the psychophysical tools of pain assessment, which rely on subjective reports—e.g., [35,36]—and provide little information about the location of perturbed nociceptive processing in the nervous system, the NWR is considered an objective physiological measure of spinal cord excitability and thus could be a valuable tool in clinical practice. However, further improvements in the methodology and studies on patients with chronic pain are needed before NWR can be implemented as an objective tool in clinical practice. The purpose of the current study was to set up an automated method for recording the NWR and to compare the NWR thresholds between patients with fibromyalgia and healthy controls.

## 2. Experimental Section

### 2.1. Study Population

The study was part of a larger effort to characterize patients with fibromyalgia. The study was granted ethical approval by the Linköping University Ethics Committee (Dnr: 2016/239-31; date of approval: 15 June 2016). All the participants gave their written informed consent, and the study was performed in accordance with the Helsinki Declaration.

Thirty-three female fibromyalgia patients and 31 age-matched female controls between 22 and 56 years of age were eligible to participate. The age-matched female controls were recruited through advertisements in newspapers, and the fibromyalgia group consisted of patients from the Pain and Rehabilitation Centre at the University Hospital in Linköping, Sweden. One patient and two controls did not show up for the experiment. In summary, 32 patients and 29 controls were studied.

During the study, five patients and one control discontinued after testing the first limb. One patient did not complete the tests on the first limb but finished the second one. In all these cases, a high level of discomfort was reported during the experiment. In one control, the result of the second limb had to be discarded due to technical issues. Accordingly, a bilateral study was conducted in 26 patients and 27 controls, and in the remaining participants only one limb was studied. Testing was performed bilaterally to examine intra-individual differences in NWR thresholds. The NWR thresholds were successfully measured in 29 patients and 21 controls.

The inclusion criteria of the patients were female gender, age 20 to 65 years, and fibromyalgia according to the 1990 ACR criteria [27]. Revised criteria for fibromyalgia were recently presented [37,38], but these were not available in the finished form when the current study was designed and ethical approval was sought. The exclusion criteria vis-à-vis chronic pain for patients and controls were rheumatoid arthritis, bursitis, tendonitis, capsulitis, postoperative conditions in the neck/shoulder area, previous neck trauma, disorder of the spine, and neurological disease. Participants who were unable to refrain from non-steroidal anti-inflammatory drugs (NSAIDs) and pain and sleep medication for 48 h prior to the visit (i.e., medical washout period) were also excluded. Other exclusion criteria were MRI incompatibility (metal in the body, claustrophobia); pregnancy; difficulty understanding Swedish; metabolic disease, including unregulated thyroid disease; severe psychiatric condition; malignancy; cardiovascular disease; lung disease; and hazardous or harmful alcohol use (≥6 Alcohol Use Disorders Identification Test (AUDIT) score [39]).

All the subjects were asked to refrain from caffeine and nicotine consumption for 12 h and heavy exercise for 48 h before the experiment. All the subjects went through a physical examination and their medical history was taken by a physician at the Pain and Rehabilitation Centre. The clinical examination ensured that the patients fulfilled the criteria for fibromyalgia according to the 1990 ACR criteria [27]. For all the participants, the height and weight were registered and body mass index calculated (BMI = weight/height^2^; kg/m^2^). Blood pressure (mm Hg) was also measured.

All the recordings were done during the daytime, either in the morning or afternoon. Both lower limbs were studied consecutively in the same experimental sitting.

### 2.2. Health Questionnaires

All the subjects answered a health questionnaire, including age (years), current pain intensity, psychological distress, disability aspects, and quality of life aspects. In the fibromyalgia group, the duration of the disease (time since diagnosis) was also noted.

The current pain intensity was registered using a numeric rating scale with anchor points 0 (denoting no pain) and 10 (denoting the worst possible pain).

The Hospital Anxiety and Depression Scale (HADS) comprises seven items in each of the two subscales: depression (HADS-D) and anxiety (HADS-A) symptoms [40,41]. Both subscale scores range from 0 to 21 and were combined to indicate the extent of psychological distress (HADS total, 0–42) [42].

The Pain Disability Index (PDI) consists of seven items, and the subjects rate how much pain interferes in these seven areas of life activity: family/home responsibilities, recreation, social activity, occupation, sexual behavior, self-care, and life-support activity [43,44]. Each area is rated using a 0 (no disability) to 10 (total disability) numeric rating scale. Scores for the all areas were combined (range, 0–70).

The European Quality of Life instrument (EQ-5D) captures a patient’s perceived state of health [45,46,47]. In the present study, the self-estimation of the current day’s health according to a 100-point scale, a thermometer-like scale (EQ-5D-VAS), was used with defined endpoints (high values indicate good health and low values indicate bad health).

### 2.3. Electrical Stimulation Procedure

Electrical stimuli were delivered to the foot with the anode attached to the mid-foot arch and the cathode attached to the dorsum of the foot. The anode was an Ambu Neuroline 700 10-J/12 silver/silver chloride self-adhesive gel electrode with a sensor area of 263 mm^2^. The cathode was an Axelgaard PALS 895,240 hydrogel self-adhesive electrode containing a stainless-steel conductive fabric with a sensor area of 4500 mm^2^. A constant current stimulator was used with a maximum output current of 16 mA at a maximum compliance voltage of 120 V (Multichannel systems MCS GmbH, Reutlingen, Germany). The two channels were connected in parallel, thus providing a maximum current of 32 mA at 120 V. The electrical stimulus composed of a train of five square wave pulses with a pulse width of 1 ms and a pulse frequency of 200 Hz—a stimulus train that is shown to effectively elicit the NWR [48,49]. The stimuli were delivered with jittered intervals of 8 to 12 s to avoid habituation [50,51]. All the subjects were naïve to the stimulation procedure.

### 2.4. Determination of the NWR Response

The NWR response was recorded on the ipsilateral tibialis anterior muscle. Three self-adhesive gel electrodes were used for active, reference, and ground recordings (3M model 2228 silver/silver chloride, sensor area 50 mm^2^; 3M, St. Paul, MN, USA). The active electrode was attached to the skin of the lower leg at a point one-third of the length of the tibialis anterior muscle from the proximal demarcation of the muscle belly palpated at isometric contraction. The reference electrode was attached to the muscle 6 cm distal to the active electrode. The ground electrode was attached to the distal lower leg between the reference electrode and the ankle. Before each test, the impedance on all the electrodes (both stimulating and recording) was noted. To reduce the electrode resistance, the skin was treated with sandpaper and washed with lukewarm water. The electrodes were connected with shielded lead wires (MLA2503 and MLA 2340) to an FE 232 amplifier and a PowerLab 4/26 data acquisition system (ADInstruments Ltd., Oxford, UK).

A software was developed in MatLab r2015b (MathWorks, Natick, MA, USA) to enable automatic NWR threshold determination based on the method suggested by Rhudy et al. [12]. The maximum amplitude recorded in a time interval of 90 to 150 ms (peak amplitude) after the start of the stimulus pulse train and the mean amplitude in the −60 to 0 ms pre-stimulus interval (baseline) were determined [12]. A Z-score was calculated as the difference between the peak amplitude and the baseline mean amplitude divided by the standard deviation of the baseline activity. A Z-score ≥ 12 was considered a successful NWR response [52].

The stimulus intensity was increased in 2 mA steps starting at 1 mA. When a successful muscle response was recorded (i.e., a Z-score ≥ 12), the intensity was decreased with steps of 1 mA until the muscle response disappeared. The stimulus intensity was then increased with increments of 0.5 mA until a second successful muscle response was recorded. Then, the intensity was decreased with steps of 0.5 mA until the response disappeared. The intensity was again increased with steps of 0.5 mA until a third muscle response appeared. The mean value of the stimulus intensities eliciting the three successful muscle reflex responses was calculated and used as the NWR threshold. The stimulation procedure continued either until three successful muscle reflex responses were detected, until the stimulus intensity reached 30 mA, or until the subject asked us to stop.

### 2.5. Subjective Pain Evaluation

Immediately after receiving each stimulus (in the 8 to 12 s interstimulus interval), the subjects were asked to rate how it felt on a graded scale ranging from 0 (no feeling) to 10 (worst imaginable pain), with four taken as the liminal value for pain and thus considered as the pain threshold (Table S1). The scale was placed before the subjects during stimulation and, in the 8 to 12 s interstimulus interval, they were asked to give a number that corresponded best to their perception of the stimulus. No further description was asked for.

### 2.6. Statistical Analysis

IBM SPSS Statistics 24 (IBM, Armonk, NY, USA) was used for a statistical analysis. Parametric descriptive statistics were used for the continuous normally distributed variables. For the non-normally distributed continuous data, ordinal and categorical variables, non-parametric descriptive statistics and procedures were used. The Shapiro-Wilk test was used to check for normality and skewness and kurtosis values >+3 or <−3 indicated a non-normal distribution. Levene’s test was used to check for the homogeneity of variance when comparing the two study groups. A Student’s *t*-test was used to assess the between-group differences of independent variables. A paired *t*-test and Wilcoxon signed-rank test were used for parametric and non-parametric paired analyses, respectively, for in-group comparisons of the 1st and 2nd limbs (NWR thresholds and pain ratings). A *p*-value <0.05 was considered significant in all the statistical analyses.

## 3. Results

### 3.1. Study Population Characteristics

The patients had on average 16 (SD 3) tender points and a mean disease duration (time from diagnosis) of 5.8 (SD 5.6) years. The BMI was significantly higher in the patient group compared to the controls (Table 1). While the controls reported no pain, the current pain intensity in patients was above 5 on a 0 to 10 numeric rating scale (Table 1). Moreover, they also reported significantly higher psychological distress (HADS total), a higher degree of disability (PDI), and a lower level of health (EQ-5D-VAS) compared to the controls (Table 1). Almost all the patients (*n* = 25) used at least one pain-relieving medication (on average 2; range 1 to 5), most commonly paracetamol (*n* = 17), but also NSAIDs (*n* = 8) and opioids (*n* = 7). Selective serotonin reuptake inhibitors (SSRIs, *n* = 7), and serotonin–norepinephrine reuptake inhibitors (SNRIs, *n* = 6) were also used as well as Amitriptyline (*n* = 9) and Gabapentin (*n* = 2). In the control group, one subject used Amitriptyline, and another used Sumatriptan. All the subjects were asked to refrain from pain-relieving medication for 48 h before the visit.

### 3.2. NWR Thresholds

The NWR thresholds were successfully measured in 29 patients and 21 controls. In 17 patients and 12 controls, two values were obtained (one from each limb), and in these cases the mean of the two values was considered as the subject’s reflex threshold. The threshold values were normally distributed in both the groups and showed a similar variance.

The mean NWR threshold did not differ between the study groups, and a large spread of the individual values was seen in both the patients and the controls (Table 2). There was no significant difference in the mean threshold between the first and second limb (Table 3). In two patients and one control, the threshold value was the same in both limbs.

No significant correlations existed between the NWR thresholds and the psychometric scales (HADS, PDI, and EQ-5D-VAS) or the number of pain-relieving medications, either in all the subjects taken together or in the two study groups.

The impedance of the stimulating electrodes remained high during the study, in particular on the electrode attached to the sole of the foot. However, no significant difference in impedance values was seen between the groups (Table 4).

### 3.3. Subjective Pain Ratings

**Figure 1:**
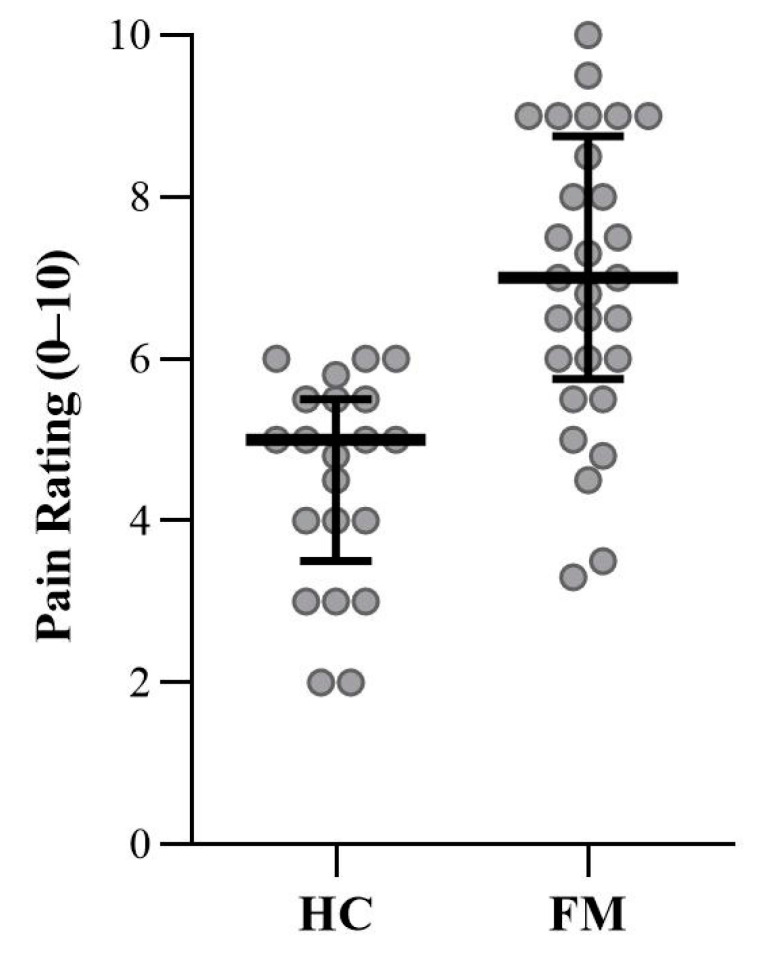
Subjective pain ratings in the fibromyalgia patients (*n* = 29) and healthy controls (*n* = 21). The figure shows individual data points for each participant (ratings across limbs were averaged) and median with interquartile range. Immediately after receiving each stimulus (in the 8 to 12 s interstimulus interval), the subjects were asked to rate how it felt on a graded scale from 0 to 10. Four was the liminal value for pain. The pain ratings were significantly higher in the patient group compared to the healthy controls (Mann–Whitney U-test: *p* < 0.001). HC, healthy controls. FM, fibromyalgia.

**Figure 2:**
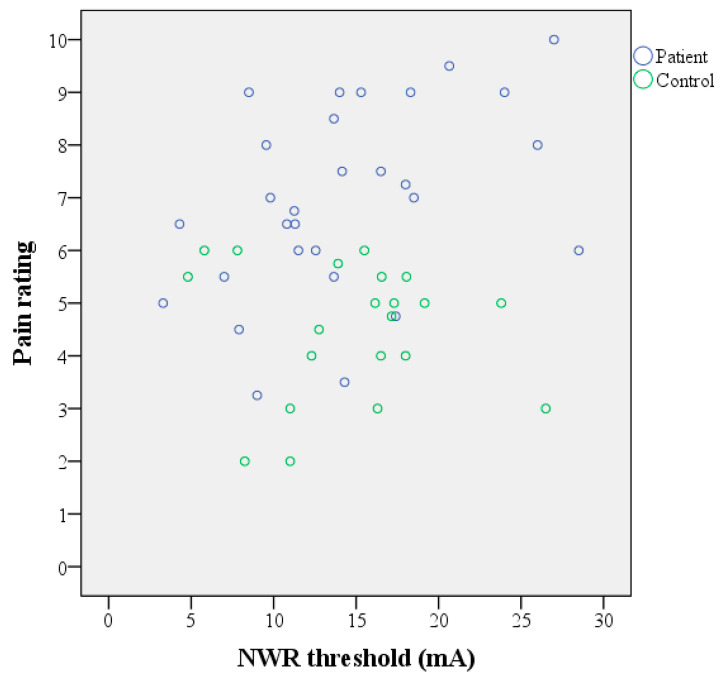
Subjective pain ratings plotted against the NWR thresholds in the fibromyalgia patients (*n* = 29) and healthy controls (*n* = 21). In subjects where NWR thresholds were obtained from both limbs, these and the corresponding pain ratings were averaged. A positive linear correlation was seen in the patient group (Spearman’s Rho = 0.460; *p* = 0.012).

The subjective pain ratings were significantly higher in the patient group compared to the healthy controls (Table 2 and Figure 1). There was no significant change in the pain ratings between the first and second limb (Table 3). Both groups received on average the same stimulus intensities (Table 4). In the patient group, a positive linear correlation between the NWR threshold and the subjective pain ratings was found (Table 2 and Figure 2). No such association was found in the control group.

## 4. Discussion

In the current study, we compared the thresholds for eliciting the NWR in fibromyalgia patients and matched healthy controls. The strength of the current study was the use of an automated and unbiased procedure for determining the NWR thresholds in a well-characterized cohort of fibromyalgia. We found no statistical difference in the NWR thresholds between the two groups. Large inter-individual variability in the NWR thresholds was seen among both patients and healthy controls. The patient group included the lowest threshold values as expected, but also somewhat surprisingly, the highest ones (Figure 2). This is consistent with other reports of a large heterogeneity in fibromyalgia [53,54,55]. Previous studies have reported reduced NWR thresholds in fibromyalgia [7,8,33], but increased thresholds have also been reported [34]. Two of these studies have small sample sizes [8,34], which limits the interpretation of the results. Besides the heterogeneity of the condition, methodological differences also exist, including the criteria for determining the presence of the NWR and calculating the thresholds. Our study is the first, to our knowledge, where the foot-to-tibialis anterior approach was used. In other studies, the sural nerve was stimulated, and recordings were made at the biceps femoris muscle. In future studies with larger sample sizes, it may be important to investigate whether different phenotypes (subgroups) of fibromyalgia exist that could explain the heterogeneity of the NWR responses. In the current study, automated measurements were successfully performed, improving the feasibility of NWR testing for potential use in clinical settings. Indeed, the development of objective tests for the assessment of patients with chronic pain and the underlying mechanisms is highly desirable to improve clinical management. In the case of NWR, further studies on patients with chronic pain, including fibromyalgia, are needed before it can be implemented as an objective tool in clinical practice.

According to the ACR criteria of 1990 [27], the diagnosis of fibromyalgia requires widespread mechanical/pressure hyperalgesia/allodynia, as indicated by at least 11 out of 18 tender points. Pain thresholds for pressure, heat, and cold are significantly altered in fibromyalgia [56,57], which are clinically often interpreted as signs of central sensitization (increased excitability and synaptic efficacy of central nociceptive pathways [58]). Central sensitization induced by primary afferent activity is considered predominantly a C-nociceptor-driven phenomenon [59], while the NWR is predominantly signaled by the myelinated nociceptors. Conditioning and test inputs can be signaled by different afferent types; for instance, following a C-fiber conditioning input (e.g., using capsaicin), the subsequent input from A-fiber activation (e.g., using pinprick) is amplified centrally, producing secondary hyperalgesia, e.g., [60]. The NWR is facilitated (drop in thresholds) following capsaicin in healthy participants [21]. It is also facilitated in several chronic pain conditions [13,61,62].

In fibromyalgia, conditioned pain modulation is dysfunctional [63], and temporal summation is facilitated [57], indicating a defective endogenous pain modulation [64]. Omic studies have indicated that not only central but also peripheral mechanisms—e.g., in muscle and plasma—contribute to the altered pain thresholds [65,66,67]. Microneurography investigations have revealed hyperexcitable C nociceptors in fibromyalgia, including a higher propensity for spontaneous activity and sensitization to mechanical stimulation [68]. Whether myelinated nociceptors, the likely afferent substrate for reflex induction, exhibit abnormal changes in fibromyalgia remains unknown.

During the NWR determination procedure, all the subjects were asked to rate how they perceived the stimuli. The patient group rated higher pain compared to the healthy controls, indicating a higher subjective pain sensitivity in fibromyalgia. Both groups received a similar level of electrical stimulus intensity, therefore this cannot explain the different degrees of pain. Increased pain sensitivity is a characteristic feature of fibromyalgia [27,31], and our study adds further evidence to this.

Several previous studies report a close correlation between the NWR threshold and the subjective pain threshold; some of them even show a linear correlation [4,5]. In the present study, a comparison between the subjective pain ratings and the NWR thresholds revealed a positive correlation in the patient group. No association between these was found in the control group. We recently reported anhedonia to pleasant touch in fibromyalgia with intact early-stage sensory processing but an altered pattern of activation in the posterior insula during the evaluation (rating) of pleasant touch and ongoing pain, suggesting the dysfunctional evaluative processing of the current experience [69]. In the current study, we found normal reflex thresholds but abnormal pain ratings in fibromyalgia, suggesting the intact spinal processing of nociceptive input but an abnormal evaluation of the current experience (pain) that involves different, hierarchically higher processes. That reflex thresholds were normal, but the pain ratings were augmented in fibromyalgia, also suggests that central sensitization may not affect all aspects of nociception equally. Using experimental models of muscle pain, a generalized skin and muscle pain-hypersensitivity (hyperalgesia/allodynia) to normally innocuous (even subperceptual) stimuli can be evoked in healthy participants [70,71], suggesting that the requisite central circuitry may already be present, and thus an elaborate anatomical reorganization may not be necessary for this to occur.

A limitation of the custom-designed software for automatic NWR threshold determination was that it did not measure the current actually delivered but rather calculated the value based on the assumption that the impedances were sufficiently low. Despite this, the NWR thresholds reported here are similar to other studies [13,14,15]. To our knowledge, none of the published studies using the surface stimulation of the foot sole have reported impedance values. Surprisingly, in situations with a high electrode impedance and thus low stimulus intensities, the test subjects still rated the stimuli as increasingly more intense. Repeated electrical stimuli can decrease the pain threshold and facilitate the perception of pain and muscle reflex response [72]. Further, the use of sandpaper on the skin (to reduce electrode resistance) could potentially sensitize the nociceptors. In our earlier work, stroking the skin with sandpaper (0.1–10 cm/s) over several trials was perceived as unpleasant but not painful (rated as naught on a pain scale), and the affective rating returned to a neutral baseline after each trial [73]. Further, in microneurography recordings, we have not observed the sensitization of nociceptors, at least for gentle shaving on unmyelinated afferents [74]. Another limitation of the current study was the relatively small number of participants, considering the large variability in the NWR thresholds and the heterogeneity of fibromyalgia [53,54]. A larger, multi-center study could be valuable to confirm our results and possibly evaluate different fibromyalgia subgroups.

In conclusion, automated NWR threshold measurements in fibromyalgia patients and healthy controls revealed no significant differences between the groups, suggesting that spinal sensitization is not a prominent feature of fibromyalgia. However, fibromyalgia patients perceived the stimuli as more painful compared to the healthy controls, suggesting abnormalities at the perceptual level.

## Figures and Tables

**Table 1:** Clinical characteristics of the two study groups.

	FM (*n* = 29)			HC (*n* = 21)			Statistic
	Mean	95% CI	SD	Mean	95% CI	SD	*p*-Value
Age (Years)	38.9	34.4, 43.4	11.7	41.2	36.2, 46.2	11.0	0.49
Blood Pressure Systolic (mm Hg)	122	117, 126	12.8	114	110, 117	8.0	0.02
Blood Pressure Diastolic (mm Hg)	81	77, 85	10.8	76	72, 80	8.4	0.10
Height (m)	1.65	1.63, 1.68	0.06	1.69	1.67, 1.72	0.06	0.03
Weight (kg)	81.2	73.3, 89.2	19.8	69.4	64.0, 74.8	11.8	0.02
BMI (kg/m^2^)	29.5	26.9, 32.1	6.5	24.3	22.6, 26.0	3.7	<0.01
Current Pain Intensity	5.5	4.7, 6.3	2.1	0.0	NA	0.0	<0.001
HADS Total	13.3	10.7, 15.8	6.5	3.8	2.2, 5.4	3.4	<0.001
PDI	37.3	32.8, 41.8	11.3	7.8	6.9, 8.7	2.0	<0.001
EQ-5D-VAS	52.7	44.9, 60.5	19.7	86.3	83.2, 89.3	6.4	<0.001

BMI, body mass index. CI, confidence interval. EQ-5D-VAS, self-estimation of the current day’s health from the European Quality of Life instrument (EQ-5D). FM, fibromyalgia. HADS total, the sum of the two subscales of the Hospital Anxiety and Depression Scale. HC, healthy controls. PDI, Pain Disability Index. SD, standard deviation.

**Table 2:** Nociceptive withdrawal reflex (NWR) thresholds and subjective pain ratings.

	A. NWR Threshold				B. Pain Rating			C. Correlation		
	Mean	95% CI	SD	*t*-Test	Median	IQR	U-Test	Rho	95% CI	*p*-Value
FM (*n* = 29)	14.4	11.9, 16.8	6.4	*p* = 0.85	7	3	*p* < 0.001	0.460	0.113, 0.707	0.012
HC (*n* = 21)	14.7	12.2, 17.2	5.4		5	2		−0.101	−0.510, 0.345	0.664
All (*n* = 50)								0.154	−0.129, 0.414	0.286

Two-tailed *t*-test. CI, confidence interval. FM, fibromyalgia. HC, healthy controls. IQR, interquartile range. Rho, Spearman’s rho. SD, standard deviation. U-test, Mann–Whitney U-test.

**Table 3:** NWR thresholds and subjective pain ratings, 1st and 2nd limb comparison.

	A. NWR Threshold (mA)				B. Pain Rating			
Group	1st Limb Mean (95% CI) [SD]	2nd Limb Mean (95% CI) [SD]	Percent Change Mean (95% CI) [SD]	*t*-Test	1st Limb Mdn (IQR)	2nd Limb Mdn (IQR)	Percent Change Mean (95% CI) [SD]	*t*-Test
FM (*n* = 17)	11.7 (8.8, 14.5) [5.5]	13.5 (10.8, 16.3) [5.3]	47 (−14, 108) [118]	*p* = 0.29	7 (3)	7 (3)	23 (−6, 51) [55]	*p* = 0.08
HC (*n* = 12)	14.2 (10.8, 17.5) [5.3]	14.1 (11.8, 16.3) [3.5]	12 (−18, 42) [47]		5 (2)	4 (3)	−6 (−24, 12) [28]	

Percent change is the group mean value of the intra-individual difference between the first and second limbs. The *t*-tests (two-tailed) refer to the between-group difference of percent change mean values. In one patient, the NWR threshold increased by 460% (3.9 SD above the mean). With this value excluded, the percent change mean was 21 (95% CI: −7, 49) [53] and *p* = 0.64 (*t*-test). CI, confidence interval. FM, fibromyalgia. HC, healthy controls. IQR, interquartile range. Mdn, median. SD, standard deviation.

**Table 4:** Electrical stimulus properties.

	A. Intensity (mA)						B. Impedance (kOhm)					
Group	*n*	1st Limb Mean (95% CI) [SD]	*t*-Test	*n*	2nd Limb Mean (95% CI) [SD]	*t*-Test	*n*	1st Limb Mean (95% CI) [SD]	*t*-Test	*n*	2nd Limb Mean (95% CI) [SD]	*t*-Test
FM	28	10.4 (8.6, 12.3) [4.8]	*p* = 0.23	24	11.9 (10.2, 13.7) [4.2]	*p* = 0.97	26	38.1 (29.8, 46.3) [20.4]	*p* = 0.73	22	32.4 (22.7, 42.1) [21.8]	*p* = 0.38
HC	21	12.1 (10.0, 14.2) [4.5]		21	11.9 (10.6, 13.2) [2.8]		20	36.0 (24.0, 47.4) [25.1]		20	38.8 (27.4, 50.1) [24.4]	

**A.** One patient discontinued testing of the first limb but completed the second one. Five patients discontinued after testing of the first limb. **B.** In two patients and two controls, impedance values were missing for either the first or the second limb. In one patient, no impedance value was available for any of the limbs. Two-tailed *t*-test. CI, confidence interval. FM, fibromyalgia. HC, healthy controls. SD, standard deviation.

## Supplementary Materials

The following are available online at https://www.mdpi.com/2077-0383/9/6/1992/s1, Table S1: Pain scale.

## References

[1] Sherrington C.S. (1910). Flexion-reflex of the limb, crossed extension-reflex, and reflex stepping and standing. J. Physiol..

[2] Kugelberg E. (1948). Demonstration of A and C fibre components in the babinski plantar response and the pathological flexion reflex. Brain.

[3] Hagbarth K.E. (1960). Spinal withdrawal reflexes in the human lower limbs. J. Neurol. Neurosurg. Psychiatry.

[4] Willer J.C. (1977). Comparative study of perceived pain and nociceptive flexion reflex in man. Pain.

[5] Chan C.W., Dallaire M. (1989). Subjective pain sensation is linearly correlated with the flexion reflex in man. Brain Res..

[6] Sandrini G., Arrigo A., Bono G., Nappi G. (1993). The nociceptive flexion reflex as a tool for exploring pain control systems in headache and other pain syndromes. Cephalalgia.

[7] Desmeules J.A., Cedraschi C., Rapiti E., Baumgartner E., Finckh A., Cohen P., Dayer P., Vischer T.L. (2003). Neurophysiologic evidence for a central sensitization in patients with fibromyalgia. Arthritis Rheum..

[8] Banic B., Petersen-Felix S., Andersen O.K., Radanov B.P., Villiger P.M., Arendt-Nielsen L., Curatolo M. (2004). Evidence for spinal cord hypersensitivity in chronic pain after whiplash injury and in fibromyalgia. Pain.

[9] Guieu R., Serratrice G., Pouget J. (1994). Counter irritation test in primary fibromyalgia. Clin. Rheumatol..

[10] Bouhassira D., Danziger N., Attal N., Guirimand F. (2003). Comparison of the pain suppressive effects of clinical and experimental painful conditioning stimuli. Brain.

[11] Peters M.L., Schmidt A.J., Van den Hout M.A., Koopmans R., Sluijter M.E. (1992). Chronic back pain, acute postoperative pain and the activation of diffuse noxious inhibitory controls (DNIC). Pain.

[12] Rhudy J.L., France C.R. (2007). Defining the nociceptive flexion reflex (NFR) threshold in human participants: A comparison of different scoring criteria. Pain.

[13] Courtney C.A., Lewek M.D., Witte P.O., Chmell S.J., Hornby T.G. (2009). Heightened flexor withdrawal responses in subjects with knee osteoarthritis. J. Pain.

[14] Jensen M.B., Biurrun Manresa J., Andersen O.K. (2015). Reliable estimation of nociceptive withdrawal reflex thresholds. J. Neurosci. Methods.

[15] Jensen M.B., Manresa J.A.B., Frahm K.S., Andersen O.K. (2013). Analysis of muscle fiber conduction velocity enables reliable detection of surface EMG crosstalk during detection of nociceptive withdrawal reflexes. BMC Neurosci..

[16] Herm C., Silbereisen V., Graf B.M., Lassen C.L. (2019). Long term reliability of nociceptive withdrawal reflex thresholds. J. Neurosci. Methods.

[17] Lloyd D.P.C. (1943). Neuron patterns controlling transmission of ipsilateral hind limb reflexes in cat. J. Neurophysiol..

[18] Hugon M., Desmedt J.E. (1973). Exteroceptive Reflexes to Stimulation of the Sural Nerve in Normal Man. New Developments in Electromyography and Clinical Neurophysiology, Volume 3: Human Reflexes, Pathophysiology of Motor Systems, Methodology of Human Reflexes.

[19] Shahani B. (1970). Flexor reflex afferent nerve fibres in man. J. Neurol. Neurosurg. Psychiatry.

[20] Ertekin C., Ertekin N., Karcioglu M. (1975). Conduction velocity along human nociceptive reflex afferent nerve fibres. J. Neurol. Neurosurg. Psychiatry.

[21] Gronroos M., Pertovaara A. (1993). Capsaicin-induced central facilitation of a nociceptive flexion reflex in humans. Neurosci. Lett..

[22] Andersen O.K., Jensen L.M., Brennum J., Arendt-Nielsen L. (1994). Evidence for central summation of C and A delta nociceptive activity in man. Pain.

[23] Willer J.C., Albe-Fessard D. (1983). Further studies on the role of afferent input from relatively large diameter fibers in transmission of nociceptive messages in humans. Brain Res..

[24] Kugelberg E., Eklund K., Grimby L. (1960). An electromyographic study of the nociceptive reflexes of the lower limb. Mechanism of the plantar responses. Brain.

[25] Wiesenfeld-Hallin Z., Hallin R.G., Persson A. (1984). Do large diameter cutaneous afferents have a role in the transmission of nociceptive messages?. Brain Res..

[26] Nagi S.S., Marshall A.G., Makdani A., Jarocka E., Liljencrantz J., Ridderstrom M., Shaikh S., O’Neill F., Saade D., Donkervoort S. (2019). An ultrafast system for signaling mechanical pain in human skin. Sci. Adv..

[27] Wolfe F., Smythe H.A., Yunus M.B., Bennett R.M., Bombardier C., Goldenberg D.L., Tugwell P., Campbell S.M., Abeles M., Clark P. (1990). The American College of Rheumatology 1990 Criteria for the Classification of Fibromyalgia. Report of the Multicenter Criteria Committee. Arthritis Rheum..

[28] Sommer C. (2010). Fibromyalgia: A clinical update. Pain Clin. Updates.

[29] Backryd E., Tanum L., Lind A.L., Larsson A., Gordh T. (2017). Evidence of both systemic inflammation and neuroinflammation in fibromyalgia patients, as assessed by a multiplex protein panel applied to the cerebrospinal fluid and to plasma. J. Pain Res..

[30] Williams D.A., Clauw D.J. (2009). Understanding fibromyalgia: Lessons from the broader pain research community. J. Pain.

[31] Sluka K.A., Clauw D.J. (2016). Neurobiology of fibromyalgia and chronic widespread pain. Neuroscience.

[32] Vierck C.J. (2006). Mechanisms underlying development of spatially distributed chronic pain (fibromyalgia). Pain.

[33] Tanwar S., Mattoo B., Kumar U., Bhatia R. (2019). Can aberrant spinal nociception be a marker of chronicity of pain in fibromyalgia syndrome?. J. Clin. Neurosci..

[34] Rhudy J.L., DelVentura J.L., Terry E.L., Bartley E.J., Olech E., Palit S., Kerr K.L. (2013). Emotional modulation of pain and spinal nociception in fibromyalgia. Pain.

[35] Samour M.S., Nagi S.S., Shortland P.J., Mahns D.A. (2017). Minocycline prevents muscular pain hypersensitivity and cutaneous allodynia produced by repeated intramuscular injections of hypertonic saline in healthy human participants. J. Pain.

[36] Mahns D.A., Nagi S.S. (2013). An investigation into the peripheral substrates involved in the tactile modulation of cutaneous pain with emphasis on the C-tactile fibres. Exp. Brain Res..

[37] Wolfe F., Clauw D.J., Fitzcharles M.A., Goldenberg D.L., Häuser W., Katz R.L., Mease P.J., Russell A.S., Russell I.J., Walitt B. (2016). 2016 Revisions to the 2010/2011 fibromyalgia diagnostic criteria. Semin. Arthritis Rheum..

[38] Arnold L.M., Bennett R.M., Crofford L.J., Dean L.E., Clauw D.J., Goldenberg D.L., Fitzcharles M.A., Paiva E.S., Staud R., Sarzi-Puttini P. (2019). AAPT Diagnostic Criteria for Fibromyalgia. J. Pain.

[39] Bergman H., Kallmen H. (2002). Alcohol use among Swedes and a psychometric evaluation of the alcohol use disorders identification test. Alcohol Alcohol..

[40] Zigmond A.S., Snaith R.P. (1983). The hospital anxiety and depression scale. Acta Psychiatr. Scand..

[41] Bjelland I., Dahl A.A., Haug T.T., Neckelmann D. (2002). The validity of the Hospital Anxiety and Depression Scale: An updated literature review. J. Psychosom. Res..

[42] LoMartire R., Ang B.O., Gerdle B., Vixner L. (2020). Psychometric properties of Short Form-36 Health Survey, EuroQol 5-dimensions, and Hospital Anxiety and Depression Scale in patients with chronic pain. Pain.

[43] Chibnall J.T., Tait R.C. (1994). The Pain Disability Index: Factor structure and normative data. Arch. Phys. Med. Rehabil..

[44] Gronblad M., Jarvinen E., Hurri H., Hupli M., Karaharju E.O. (1994). Relationship of the Pain Disability Index (PDI) and the Oswestry Disability Questionnaire (ODQ) with three dynamic physical tests in a group of patients with chronic low-back and leg pain. Clin. J. Pain.

[45] Brooks R., Group E. (1996). EuroQol: The current state of play. Health Policy.

[46] Dolan P., Sutton M. (1997). Mapping visual analogue scale health state valuations onto standard gamble and time trade-off values. Social Sci. Med..

[47] (1990). EuroQol—A new facility for the measurement of health-related quality of life. Health Policy.

[48] Toorring J., Pedersen E., Klemar B. (1981). Standardisation of the electrical elicitation of the human flexor reflex. J. Neurol. Neurosurg. Psychiatry.

[49] Meinck H.M., Kuster S., Benecke R., Conrad B. (1985). The flexor reflex—Influence of stimulus parameters on the reflex response. Electroencephalogr. Clin. Neurophysiol..

[50] Faganel J. (1970). An analysis of flexor reflex elicited by rhythmic and stochastic stimulation in normal man. Jugoslav. Physiol. Pharmacol. Acta.

[51] Dimitrijevic M.R., Faganel J., Gregoric M., Nathan P.W., Trontelj J.K. (1972). Habituation: Effects of regular and stochastic stimulation. J. Neurol. Neurosurg. Psychiatry.

[52] France C.R., Rhudy J.L., McGlone S. (2009). Using normalized EMG to define the nociceptive flexion reflex (NFR) threshold: Further evaluation of standardized NFR scoring criteria. Pain.

[53] Hurtig I.M., Raak R.I., Kendall S.A., Gerdle B., Wahren L.K. (2001). Quantitative sensory testing in fibromyalgia patients and in healthy subjects: Identification of subgroups. Clin. J. Pain.

[54] Yim Y.R., Lee K.E., Park D.J., Kim S.H., Nah S.S., Lee J.H., Kim S.K., Lee Y.A., Hong S.J., Kim H.S. (2017). Identifying fibromyalgia subgroups using cluster analysis: Relationships with clinical variables. Eur. J. Pain.

[55] Bartley E.J., Robinson M.E., Staud R. (2018). Pain and Fatigue Variability Patterns Distinguish Subgroups of Fibromyalgia Patients. J. Pain.

[56] Palmer S., Bailey J., Brown C., Jones A., McCabe C.S. (2019). Sensory Function and Pain Experience in Arthritis, Complex Regional Pain Syndrome, Fibromyalgia Syndrome, and Pain-Free Volunteers: A Cross-Sectional Study. Clin. J. Pain.

[57] Goubert D., Danneels L., Graven-Nielsen T., Descheemaeker F., Meeus M. (2017). Differences in Pain Processing Between Patients with Chronic Low Back Pain, Recurrent Low Back Pain, and Fibromyalgia. Pain Physician.

[58] Woolf C.J. (2011). Central sensitization: Implications for the diagnosis and treatment of pain. Pain.

[59] Mendell L.M., Wall P.D. (1965). Responses of single dorsal cord cells to peripheral cutaneous unmyelinated fibres. Nature.

[60] Treede R.D., Cole J.D. (1993). Dissociated secondary hyperalgesia in a subject with a large-fibre sensory neuropathy. Pain.

[61] Filatova E., Latysheva N., Kurenkov A. (2008). Evidence of persistent central sensitization in chronic headaches: A multi-method study. J. Headache Pain.

[62] Sterling M., Hodkinson E., Pettiford C., Souvlis T., Curatolo M. (2008). Psychologic factors are related to some sensory pain thresholds but not nociceptive flexion reflex threshold in chronic whiplash. Clin. J. Pain.

[63] Gerhardt A., Eich W., Treede R.D., Tesarz J. (2017). Conditioned pain modulation in patients with nonspecific chronic back pain with chronic local pain, chronic widespread pain, and fibromyalgia. Pain.

[64] O’Brien A.T., Deitos A., Triñanes Pego Y., Fregni F., Carrillo-de-la-Peña M.T. (2018). Defective endogenous pain modulation in fibromyalgia: A meta-analysis of temporal summation and conditioned pain modulation paradigms. J. Pain.

[65] Olausson P., Ghafouri B., Ghafouri N., Gerdle B. (2016). Specific proteins of the trapezius muscle correlate with pain intensity and sensitivity—An explorative multivariate proteomic study of the trapezius muscle in women with chronic widespread pain. J. Pain Res..

[66] Gerdle B., Ghafouri B., Ghafouri N., Bäckryd E., Gordh T. (2017). Signs of ongoing inflammation in female patients with chronic widespread pain: A multivariate, explorative, cross-sectional study of blood samples. Medicine (Baltimore).

[67] Gerdle B., Wåhlén K., Ghafouri B. (2020). Plasma protein patterns are strongly correlated with pressure pain thresholds in women with chronic widespread pain and in healthy controls-an exploratory case-control study. Medicine (Baltimore).

[68] Serra J., Collado A., Solà R., Antonelli F., Torres X., Salgueiro M., Quiles C., Bostock H. (2014). Hyperexcitable C nociceptors in fibromyalgia. Ann. Neurol..

[69] Boehme R., van Ettinger-Veenstra H., Olausson H., Gerdle B., Nagi S.S. (2020). Anhedonia to gentle touch in fibromyalgia: Normal sensory processing but abnormal evaluation. Brain Sci..

[70] Nagi S.S., Mahns D.A. (2013). Mechanical allodynia in human glabrous skin mediated by low-threshold cutaneous mechanoreceptors with unmyelinated fibres. Exp. Brain Res..

[71] Dunn J.S., Nagi S.S., Mahns D.A. (2019). Regionally diffuse muscle pain-hypersensitivity in humans during acute muscle pain. bioRxiv.

[72] Arendt-Nielsen L., Brennum J., Sindrup S., Bak P. (1994). Electrophysiological and psychophysical quantification of temporal summation in the human nociceptive system. Eur. J. Appl. Physiol. Occup. Physiol..

[73] Shaikh S., Nagi S.S., McGlone F., Mahns D.A. (2015). Psychophysical investigations into the role of low-threshold C fibres in non-painful affective processing and pain modulation. PLoS ONE.

[74] Vallbo Å.B., Olausson H., Wessberg J. (1999). Unmyelinated afferents constitute a second system coding tactile stimuli of the human hairy skin. J. Neurophysiol..

