# Supplementary material for: Automated Nociceptive Withdrawal Reflex Measurements Reveal Normal Reflex Thresholds and Augmented Pain Ratings in Patients with Fibromyalgia
Source: J Clin Med. 2020 Jun 25;9(6):1992. doi: 10.3390/jcm9061992 (PMC7356211; doi:10.3390/jcm9061992)
Supplement: Supplementary file 1 [file jcm-09-01992-s001.pdf]

Supplementary Material

The scale used by the study participants to rate their subjective pain perception during the NWR determination procedure. Immediately after receiving each stimulus (in the 8 to 12 s interstimulus interval), the subjects were asked to rate how it felt. The subjects had the scale placed before them during the recording and were asked to give a number that corresponded best to their perception of the stimulus. No further description was asked for. All subjects understood the Swedish language well. The English translation is for presentation purposes.

Table S1. Pain scale

	**Intensitet**	**Intensity**
**0**	Ingen känselförnimmelse	No feeling
**1**	Svag känselförnimmelse	Slight feeling
**2**	Tydlig känselförnimmelse	Distinct feeling
**3**	Obehag	Unpleasantness
**4**	Mycket svag smärta	Just noticeable pain
**5**	Svag smärta	Slight pain
**6**	Tydlig smärta	Distinct pain
**7**	Måttligt stark smärta	Moderately intense pain
**8**	Stark smärta	Intense pain
**9**	Mycket stark smärta	Very intense pain
**10**	Värsta tänkbara smärta	Worst imaginable pain
